# Epigenetic chaperoning of aging

**DOI:** 10.18632/aging.102808

**Published:** 2020-01-28

**Authors:** Stefanie Müthel, Baris Tursun

**Affiliations:** 1 Berlin Institute of Medical Systems Biology, 10115 Berlin, Germany; 2 Max Delbrück Center for Molecular Medicine in the Helmholtz Association, 13125 Berlin, Germany

**Keywords:** aging, healthspan, epigenetic, histone chaperone, metabolism

Who wants to live forever? Lifespan extension is a long-standing human desire. And sure enough, a longer lifespan is expected to also extend the time without diseases and frailty. But should we take for granted that lifespan and healthspan are inherently linked? Meaning, does increased lifespan also extend the time without unfavorable health conditions? It is not well understood to which extend lifespan and healthspan are indeed linked. Recent studies provide increasing evidence that epigenetic factors may play a pivotal role in connecting aging and healthspan regulation.

Epigenetic factors control gene expression by regulating modifications and structure of chromatin. Loss of specific chromatin regulators can cause defects and diseases such as cancer and neurodegeneration. Also, histone demethylases maintain muscle stem cells and Sirtuins with histone deacetylation activity control organismal aging [1]. Another type of chromatin-regulating proteins are histone chaperones. They are required for nuclear import of histone proteins, their assembly into nucleosomes and genomic localization, as well as for the post-translational modifications of histones. Histone chaperones interact with different chromatin-regulating factors to provide gene regulatory functions with a broad spectrum of physiological functions such as cell fate safeguarding and blocking reprogramming of cell identities [2].

Recently, it was found that the histone chaperone LIN-53 in the nematode *Caenorhabditis elegans* (*C. elegans*), known as RBBP4 and RBBP7 in humans, is important for lifespan as well as healthspan regulation [3]. Interestingly, a previous study in humans revealed that RBBP4/7 are implicated in aging and age-related memory loss [4,5]. Therefore, it is conceivable that LIN-53 represents and evolutionarily conserved link of lifespan and healthspan regulation.

The histone chaperone LIN-53 and its mammalian homologs RBBP4/7 (older names: CAF-1p48, RbAp46/48) are found in different chromatin-regulating complexes including Polycomb Repressive Complex 2 (PRC2, histone methyltransferase activity), CAF1 histone chaperone complex, Sin3 histone deacetylase complex (HDAC), nucleosome remodeling complex (NuRD), and DRM (Dp/Rb/Muv) complex [6]. Recently, LIN-53 and the RBBP4/7-containing CAF1 complex have been identified as reprogramming barriers in the nematode *C. elegans* and in mouse fibroblasts, respectively. Additionally, LIN-53 and its homologs play important roles during cell cycle regulation in *C. elegans*, *Drosophila*, and mammals [7] pointing to an evolutionarily conserved repertoire of different physiological functions.

LIN-53 links lifespan and healthspan. Loss of LIN-53 in *C. elegans* leads to a severe decrease in muscle function and a shortened lifespan [3]. Motility defects due to deteriorated muscle structure of *lin-53* mutant animals are apparent in developing and aging animals. Genetic and biochemical analysis revealed that LIN-53’s role in maintaining muscle health functions through the chromatin-remodeling complex NuRD. Interestingly, loss of other NuRD component cause muscle defects without affecting lifespan, indicating that LIN-53 depletion causes two separable phenotypes: a short lifespan and a short healthspan.

Indeed, LIN-53’s role in regulating aging could be attributed to its function through the HDAC complex Sin3, which causes a shortened lifespan also in *Drosophila* when depleted [3]. Analogously to the NuRD conundrum, depletion of Sin3 decreases lifespan but animals do not show obvious muscle health defects during aging [3]. Multi-layered omics analysis revealed that loss of either LIN-53 or Sin3 leads to changes in the expression of genes regulating metabolic pathways, and that LIN-53 binds directly to these genes [3]. Metabolomics of *lin-53* and *sin-3* mutants pointed to a marked decrease of trehalose levels - a disaccharide required for normal lifespan in worms [8]. Reduced trehalose levels are caused by impaired expression of genes required for trehalose synthesis. They are directly regulated by DAF-16 - a FOXO transcription factor which regulates aging and acts downstream of the insulin/IGF-pathway [8]. In LIN-53-depleted animals DAF-16 translocation from the cytoplasm to the nucleus is diminished, thereby causing insufficient gene expression for trehalose synthesis. Notably, increasing trehalose levels of *lin-53* mutants by trehalose feeding rescued the short lifespan but not the muscle defects [3], which corroborates the notion that regulation of aging and healthspan can be separated. Overall, LIN-53 epigenetically combines healthspan maintenance through the NuRD complex with aging regulation via the HDAC Sin3.

Such epigenetic link may have been conserved in evolution. The human LIN-53 homologs RBBP4 and RBBP7 are implicated in the early childhood aging disease Hutchinson-Gilford Progeria Syndrome (HGPS) [4]. HGPS patients carry a mutation in the lamin A/C gene encoding for the nuclear envelope protein lamin. Patient cells show low RBBP4/7 protein levels and loss of RBBP4/7 association with the nuclear lamina causes chromatin mis-regulation. HGPS belongs to laminopathy disorders, which also include Emery–Dreifuss muscular dystrophy (EDMD). EDMD patients suffer from diminished muscle maintenance and it is possible that RBBP4/7 are generally affected in such laminopathies, which would recapitulate the short lifespan and muscle phenotypes in worms (Figure 1). Overall, LIN‐53 and RBBP4/7 may epigenetically connect lifespan and healthspan maintenance in *C. elegans* as well as humans. This notion is also supported by a recent study revealing that the decline of RBBP4 in humans causes cognitive aging and memory loss [5]. It is therefore conceivable that LIN‐53 and its homologs represent an evolutionarily conserved link of lifespan and healthspan regulation across species.

**Figure 1:**
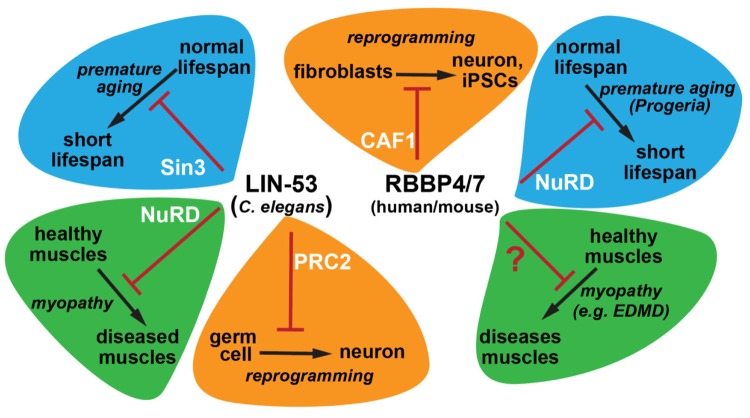
**Physiological functions of the histone chaperone homologs LIN-53 and RBBP4/7 with respect to cellular safeguarding, muscular healthspan, and aging.** In *C. elegans* the histone chaperone LIN-53 functions through the histone deacetylase complex Sin3 to ensure normal lifespan, while its function via the chromatin remodelling complex NuRD is required for muscle maintenance during aging. RBBP4/7 are the human homologs of LIN-53 and act through NuRD to prevent premature aging as seen in patients with the laminopathy disease Progeria. It remains to be determined whether RBBP4/7 are also required for muscle healthspan to prevent muscle decay in laminopathies with known muscle phenotypes. LIN-53 and its homologs safeguard cell fates by acting as reprogramming barriers via the PRC2 complex in *C. elegans* and CAF1 complex in mouse fibroblasts.

In the future further studies to elucidate the molecular links of lifespan and healthspan regulation based on epigenetics and metabolism will be required to better understand the relevant aspects of aging. But: Who waits forever anyway?

## References

[1] Brunet A, Rando TA. Curr Opin Cell Biol. 2017; 45:1–7. 10.1016/j.ceb.2016.12.009 28129586 PMC5482778

[2] Kolundzic E, et al. Dev Cell. 2018; 46:611–626.e12. 10.1016/j.devcel.2018.07.006 30078731 PMC6137076

[3] Müthel S, et al. Aging Cell. 2019; 18:e13012. 10.1111/acel.13012 31397537 PMC6826145

[4] Pegoraro G, et al. Nat Cell Biol. 2009; 11:1261–67. 10.1038/ncb1971 19734887 PMC2779731

[5] Pavlopoulos ES, et al. Science Translational Medicine. 2013; 5:200ra115. 10.1126/scitranslmed.3006373 23986399 PMC4940031

[6] Seelk S, et al. eLife. 2016; 7:27602485. 10.7554/eLife.15477 27602485 PMC5045294

[7] Fischer M, Müller GA. Crit Rev Biochem Mol Biol. 2017; 52:638–62. 10.1080/10409238.2017.1360836 28799433

[8] Seo Y, et al. Proc Natl Acad Sci USA. 2018; 115:E2791–800. 10.1073/pnas.1714178115 29511104 PMC5866546

